# Supplementary material for: Assessment of hemagglutinin-inhibition activity following influenza vaccination during the 2022–2023, 2023–2024, and 2024–2025 seasons
Source: PLoS One. 2026 Apr 16;21(4):e0347314. doi: 10.1371/journal.pone.0347314 (PMC13086323; doi:10.1371/journal.pone.0347314)
Supplement: S1 Table — (DOCX) [file pone.0347314.s001.docx]

| **Vaccine type (UGA7>UGA8>UGA9)** | **Number of recipients** |
| --- | --- |
| FZ SD>FZ SD>FZ SD | 59 |
| FZ HD>FZ HD>FZ HD | 54 |
| FM>FM>FM | 16 |
| FC>FC>FC | 15 |
| FZ HD > FA > FA | 13 |
| FB > FC > FB | 8 |
| FB > FZ SD > FZ SD | 6 |
| FZ SD > FZ HD > FZ HD | 5 |
| FZ SD > FC > FC | 5 |
| FC > FZ SD > FZ SD | 4 |
| FZ SD>FZ SD>FB | 3 |
| FB > FM > FM | 3 |
| FZ SD > FZ SD > FZ HD | 2 |
| FB > FC > FC | 2 |
| FZ SD > FA > FA | 1 |
| FZ SD> FZ HD > FZ HD | 1 |
| FB>FZ SD>FB | 1 |
| FZ SD > FC > FB | 1 |
| FB>FC>FB | 1 |
| FC>FC>FZ HD | 1 |
| FM > FZ SD > FZ SD | 1 |
| FZ HD>FA>FA | 1 |
| **Total** | **203** |

**Table S1.** Vaccine type and number of vaccine recipients during 2022-2023, 2023-2024, and the 2024-2025 influenza seasons.

FZ SD: Fluzone Standard Dose, FZ HD: Fluzone High Dose, FM: Flumist, FB: Flublok, FC: Flucelvax, FA: Fluad.
