# Supplementary material for: Assessment of hemagglutinin-inhibition activity following influenza vaccination during the 2022–2023, 2023–2024, and 2024–2025 seasons
Source: PLoS One. 2026 Apr 16;21(4):e0347314. doi: 10.1371/journal.pone.0347314 (PMC13086323; doi:10.1371/journal.pone.0347314)
Supplement: Attachment — Submitted filename: Berber PLoS One Response to Reviewers.docx [file pone.0347314.s009.docx]

March 8, 2026

Patricia Evelyn Fast, MD, Ph.D.

Academic Editor

PLOS One

**Response to Reviewers and Academic Editor**

**Manuscript ID:** PONE-D-25-48769
**Title:** Assessment of Hemagglutinin-Inhibition Activity Following Influenza Vaccination During the 2022-2023, 2023-2024, and 2024-2025 Seasons
**Journal:** PLOS ONE

**Journal Requirements: 1.**

When submitting your revision, we need you to address these additional requirements.

 Please ensure that your manuscript meets PLOS ONE's style requirements, including those for file naming. The PLOS ONE style templates can be found at

[https://journals.plos.org/plosone/s/file?id=wjVg/PLOSOne_formatting_sample_main_body.pdf](https://nam04.safelinks.protection.outlook.com/?url=https%3A%2F%2Fjournals.plos.org%2Fplosone%2Fs%2Ffile%3Fid%3DwjVg%2FPLOSOne_formatting_sample_main_body.pdf&data=05%7C02%7Ceberbe1%40lsu.edu%7Cca5ce2a032a141e4a43108de5da3aa17%7C2d4dad3f50ae47d983a09ae2b1f466f8%7C0%7C0%7C639051155148452596%7CUnknown%7CTWFpbGZsb3d8eyJFbXB0eU1hcGkiOnRydWUsIlYiOiIwLjAuMDAwMCIsIlAiOiJXaW4zMiIsIkFOIjoiTWFpbCIsIldUIjoyfQ%3D%3D%7C0%7C%7C%7C&sdata=dPjrlDdL2dEJnsnaZkUbFGAPRA1LW1PzlxilJsb4EAA%3D&reserved=0) and

[https://journals.plos.org/plosone/s/file?id=ba62/PLOSOne_formatting_sample_title_authors_affiliations.pdf](https://nam04.safelinks.protection.outlook.com/?url=https%3A%2F%2Fjournals.plos.org%2Fplosone%2Fs%2Ffile%3Fid%3Dba62%2FPLOSOne_formatting_sample_title_authors_affiliations.pdf&data=05%7C02%7Ceberbe1%40lsu.edu%7Cca5ce2a032a141e4a43108de5da3aa17%7C2d4dad3f50ae47d983a09ae2b1f466f8%7C0%7C0%7C639051155148472958%7CUnknown%7CTWFpbGZsb3d8eyJFbXB0eU1hcGkiOnRydWUsIlYiOiIwLjAuMDAwMCIsIlAiOiJXaW4zMiIsIkFOIjoiTWFpbCIsIldUIjoyfQ%3D%3D%7C0%7C%7C%7C&sdata=6VpH6%2B9tYP06tpeV9REAUwMJXE7tn7NUs%2FdjcHI69G8%3D&reserved=0)

**Author response:** We formatted the title page and main text to match the PLOS ONE style templates, and ensure all submission file names follow PLOS ONE conventions (e.g., Manuscript, Revised Manuscript with Track Changes, Response to Reviewers, Figures, Supporting Information).

**Journal Requirements: 2.**

We note that the grant information you provided in the ‘Funding Information’ and ‘Financial Disclosure’ sections do not match.

**Author response:** We reconciled and made the grant/contract numbers consistent across (i) the manuscript Funding section and (ii) the online submission ‘Funding Information’ and ‘Financial Disclosure’ fields.

**Journal Requirements: 3.**

Thank you for stating the following financial disclosure:

This project has been funded by the National Institute of Allergy and Infectious Diseases, a component of the NIH, Department of Health and Human Services, under contract 75N93019C00052

Please state what role the funders took in the study.  If the funders had no role, please state: "The funders had no role in study design, data collection and analysis, decision to publish, or preparation of the manuscript."

If this statement is not correct you must amend it as needed.

Please include this amended Role of Funder statement in your cover letter; we will change the online submission form on your behalf.

**Author response:** We confirm that “The funders had no role in study design, data collection and analysis, decision to publish, or preparation of the manuscript.” Please do not hesitate to update the online submission fields on our behalf to reflect the following funding information:

TMR: Contract 75N93019C00052; National Institute of Allergy and Infectious Diseases (NIAID), National Institutes of Health (NIH), U.S. Department of Health and Human Services; <https://www.niaid.nih.gov/> and <https://www.nih.gov/>

TMR: GRA-001; Georgia Research Alliance; <https://gra.org/>

**************************************************************************

During submission:

In the manuscript draft:

***************************************************************************

**Journal Requirements: 4.**

When completing the data availability statement of the submission form, you indicated that you will make your data available on acceptance. We strongly recommend all authors decide on a data sharing plan before acceptance, as the process can be lengthy and hold up publication timelines. Please note that, though access restrictions are acceptable now, your entire data will need to be made freely accessible if your manuscript is accepted for publication. This policy applies to all data except where public deposition would breach compliance with the protocol approved by your research ethics board. If you are unable to adhere to our open data policy, please kindly revise your statement to explain your reasoning and we will seek the editor's input on an exemption. Please be assured that, once you have provided your new statement, the assessment of your exemption will not hold up the peer review process.

**Author response:** Thanks for your attention. Now, we made all data underlying the findings are provided in the Supporting Information files (S1 Data–S8 Data).

**Journal Requirements: 5.**

Please upload a new copy of Figures 1 – 9, as the detail is not clear. Please follow the link for more information:  [https://journals.plos.org/plosone/s/figures](https://nam04.safelinks.protection.outlook.com/?url=https%3A%2F%2Fjournals.plos.org%2Fplosone%2Fs%2Ffigures&data=05%7C02%7Ceberbe1%40lsu.edu%7Cca5ce2a032a141e4a43108de5da3aa17%7C2d4dad3f50ae47d983a09ae2b1f466f8%7C0%7C0%7C639051155148493600%7CUnknown%7CTWFpbGZsb3d8eyJFbXB0eU1hcGkiOnRydWUsIlYiOiIwLjAuMDAwMCIsIlAiOiJXaW4zMiIsIkFOIjoiTWFpbCIsIldUIjoyfQ%3D%3D%7C0%7C%7C%7C&sdata=l%2BVti3jz8Ztgxv%2F9etK7gkVavE76Asj2uGqnAvWbSIo%3D&reserved=0)

**Author response:** We provided Figures 1–9 at publication-quality resolution and confirmed that text, axes, and symbols remain legible at final size, consistent with PLOS ONE figure specifications.

**Journal Requirements: 6.**

If the reviewer comments include a recommendation to cite specific previously published works, please review and evaluate these publications to determine whether they are relevant and should be cited. There is no requirement to cite these works unless the editor has indicated otherwise.

**Author response:** No specific citation recommendations were provided by the reviewer in the comments received. We nonetheless reviewed the relevant literature and added citations where they directly support or contextualize our findings.

**Additional Editor Comments:**

Please find a number of comments to your paper, almost all of which are aimed to improve its clarity. Please carefully review and respond to these.

Dear Editor.

Thank you for your careful review and for the constructive comments provided on our manuscript. We appreciate the editor’s and reviewers’ efforts to improve the clarity and presentation of this work. We have carefully revised the manuscript throughout in response to these comments. Specifically, we clarified the primary study questions and the scope of inference appropriate for this observational study, expanded and revised figure legends and table footnotes so they can stand alone, corrected and refined the relevant Results and Discussion text, and addressed points of wording, interpretation, and methodological clarity raised in the review.

In addition, during preparation of the revised manuscript, we carefully re-verified the dataset used to generate Table 4 against the original Excel source files. During this verification step, we identified discrepancies that arose during manual transfer of values from the analysis spreadsheet into the manuscript table. To ensure complete accuracy, we rechecked the entire dataset and regenerated Table 4 directly from the verified data. The table has now been corrected, and the corresponding text in the Results and Discussion sections has been revised to ensure consistency with the updated values.

These revisions do not affect the overall conclusions of the study but improve the accuracy and clarity of the reported results. We appreciate the opportunity to carefully review and correct this section during the revision process. All relevant data used in the tables and figures have been supplemented.

**Major comment:**

It’s difficult for an observational study with a huge amount of data but try to emphasize what the questions were that the study hoped to answer and whether it could answer them. For example, how do potential vaccines for elderly compare in antibody generating capacity? Limitation: Flublok not used in elderly.
Example: what is the effect of skipping years? There might be better responses after a skipped year, but also more vulnerability during that skipped year. (recognizing that a study such as this could not provide sufficient data to call into question a standing recommendation.)

**Author response:** We have revised the manuscript to explicitly state the primary study questions in the end of Introduction and refined the Discussion around these questions addressing these questions.

We also added a clear limitation noting that Flublok was not administered to participants ≥65 years in this cohort, which constrains comparative conclusions in older adults. Finally, we clarified what our observational data can and cannot infer regarding “skipped years”: we describe associations between prior-season vaccination history and baseline titers and post-vaccination fold-rise, but the study design does not quantify the clinical tradeoff between vulnerability during a gap year and any potential boosting after a vaccination gap, and it does not support changes to vaccination recommendations.

Please see Lines 89–95 (study objectives in Introduction)

Lines 782–787 (discussion of skipped vaccination years)

Lines 930–939 (limitations regarding Flublok use in elderly)

**Major comment:**
It seems that the data describing the tables and graphs is in the text rather than in Legends/Footnotes. Each table and graph should be able to stand alone and be interpretable. Sometimes, they are not clearly enough labelled to do so.

Please clarify as much as possible in Table/Figure and text when data relate to the repeater cohort and when to the overall population.

**Author response:** We have revisited all figure and table legends to ensure that each figure/table is interpretable without relying on the main text. We moved essential definitions into the legends/footnotes, including D0 vs D28, the seroprotection threshold, the seroconversion definition, cohort definitions, sample sizes, and statistical tests. We also clarified, for each figure/table, whether the data are derived from the overall seasonal cohorts or the longitudinal repeater cohort.

**Additional Editor Comments: Minor comments**

**Minor comment (Abstract):**

Abstract:
Clarify whether the lower titers in elderly people were in fact a factor in the calculated higher seroconversion rates, i.e. by allowing individuals to regain seronegative status prior to the next year’s vaccination, were more seroconversions (which require seronegativity??), made possible?

**Author response:** We made it clear in the Abstract that higher seroconversion rates in older adults can be driven by lower baseline (D0) titers, because the seroconversion definition depends on a ≥4-fold rise and a post-vaccination threshold. We also avoid implying that “seronegativity” is required beyond the formal definition used. Lines 42-45.

**Minor comment:**

For the Flublok vs Hi dose FluZone comparison, state whether ages were comparable in the 2 groups. An age disparity would be a limitation on comparing the vaccines.

**Author response:** Age distributions were not comparable between Flublok and Fluzone High-Dose recipients. Fluzone HD was administered almost exclusively to participants aged ≥65 years (mean age ~73–74 years), whereas Flublok recipients were predominantly younger adults (mean age ~43–45 years). This age imbalance represents a limitation for direct between-vaccine comparisons, as age is a major determinant of HAI responses. We set this major limitation of this study and have been added to the discussion. Lines 930-938.

**Minor comment (Abstract):**

Line 46-8 “…serum HAI activity was similar in elderly participants 47 following vaccination with either Fluzone HD or Fluad against both influenza A or B vaccine 48 components”. Please clarify what is being compared here; is it FluA and FluB titers after the two vaccines mentioned? Or is there a broader comparison here?

**Author response:** We revised the sentence to clarify that the comparison is specifically between Fluzone HD and Fluad recipients in elderly participants, and that post-vaccination HAI titers are compared for each vaccine component (influenza A and influenza B strains). These results can be found in Figs 8 and 9. Please see lines 48-50.

**Minor comment (Methods):**

No vaccine in the 4 weeks since last vaccination seems an unusual criterion, since people of this age would not be expected to receive two influenza vaccines only 4 weeks apart. Is there some rationale that could be explained?

**Author response:** We removed the “within 4 weeks” exclusion criterion. Instead, eligibility required that participants had not yet received the seasonal influenza vaccine in that influenza season. We also clarified that concurrent participation in another influenza vaccine study was an exclusion criterion to avoid overlapping vaccinations or study interventions that could confound immunogenicity outcomes. Lines 97-98.

Table 2 Footnote. IAV (H1N1) strains are selected to reflect circulating (missing word?)

**Author response:** Thank you for noting this. We corrected the Table 2 footnote to include the missing word and to clarify the wording: “….reflect circulating viruses”. 119-125

**Minor comment (Table 3):**

If an individual in the repeater cohort moved from one age category to the next over 3 years, how were the data handled? Is Table 3 the original age or is the actual age in that year used?

**Author response:** Table 3 summarizes demographics for the longitudinal repeater cohort at enrollment (baseline) for descriptive purposes. We have added “Age and BMI represent measurements at first enrollment (2022–2023 season) for the longitudinal repeater cohort” to the Table 3 footnote. Line 144.

**Minor comment (Discussion):**

Line 210. In discussion, consider effect of overall greater age?

**Author response:** We have added a new paragraph in the Discussion (lines 723-729) to explicitly address the demographic confounding introduced by the increasing mean age of our enrolled population across the three seasons (from 47.1 years to 52.4 years in 2024–2025). Lines 720-733.

**Minor comment:**

Line 212. The proportion of adolescents (≤17 years) was relatively small in the 2022-2023 season (9.5%) 213 and 2024-2025 season (5.3%) but increased to 6.5% in 2023-2024 season. The text seems to imply the last season was greater than the first, but in fact first season is greatest. Consider rewording?

**Author response:** Thank you for pointing this out. We revised the text to clarify the temporal trend across seasons and to avoid any unintended implication that the later season exceeded the earlier one. The revised sentence now states that the proportion of adolescents (≤17 years) decreased from 9.5% in 2022–2023 to 6.5% in 2023–2024 and further to 5.3% in 2024–2025. Lines 240-243.

**Minor comment:**

Line 239. Consider stating ages receiving FluMist, and whether recipients had previously been vaccinated with IAV. Prior HAI titer might interfere with take.

**Author response:** FluMist recipients were primarily younger participants, consistent with the licensed age indication (2–49 years). Line 267-269.

Prior HAI titers from prior IAV vaccination may interfere with LAIV “take” by limiting vaccine virus replication and shedding in primed individuals, which could contribute to the low measurable serum HAI responses observed after LAIV administration due to neutralization. In our repeater cohort, FluMist was administered to 16 participants across all three seasons (FM>FM>FM).

Within the study period, only three repeaters received Flublok (recombinant IAV) in UGA7 followed by FluMist in UGA8 and UGA9 (FB>FM>FM). Because detailed influenza vaccination history prior to study enrollment was not consistently available for all FluMist recipients, we were unable to comprehensively stratify FluMist outcomes by prior IAV vaccination status beyond the within-study repeat-vaccination patterns described.

**Minor comment (Question):**

Question: were all vaccinations given according to manufacturers age recommendations/indications?

**Author response:** All vaccines were administered according to licensed manufacturer age indications and FDA-approved, age-based recommendations, as reflected by the age distributions for each vaccine type in our cohort (FluMist: 2–49 years; Fluzone SD/HD: ≥6 months/≥65 years; Flucelvax: ≥6 months; Flublok: ≥18 years; Fluad: ≥65 years). We have included this statement: “All vaccines were administered according to licensed manufacturer age indications and FDA-approved, age-based recommendations.” in our revised draft: 244-246.

**Minor comment (FIGURE 1):**

Needs a legend, which I did not find. Is it correct that the first column is D0 and the second D28 under each strain?

**Author response:** Figure legends are placed after the first paragraph where the figure is cited. We have revised figure legends with details as requested, and have stated “Within each strain, paired columns represent D0 (left) and D28 (right)”. Line 284-301.

**Minor comment (FIGURE 2):**

It’s not easy to follow the rise in titer within each age group for each year. The lines connect baseline to baseline and post-vaccination to post-vaccination, which does not seem like the primary focus. Perhaps all values could be on a 0, 28 day timeline with before and after vaccination linked by a line. Then the confidence intervals could be displayed.

**Author response:** Thank you for this helpful suggestion. We have revised Figure 2 to improve readability and to emphasize within-season vaccine-associated changes. Specifically, we now display values on a 0/28-day timeline within each season, and D0 and D28 are linked by lines within each season for each age group to facilitate visual comparison of pre- versus post-vaccination titers. We also added 95% confidence intervals to the plotted means (error bars) and clarified the plotting approach in the updated Figure 2 legend. We believe this revision allows readers to readily assess the magnitude of the D0→D28 change within each age group for each season. Please see line 370-383 and revised Figure 2.

**Minor comment:**

Line 242. “Table in S1 Table summarizes” wording is awkward

**Author response:** We revised the sentence to remove the redundant wording. Line 272

**Minor comment:**

Line 270 “had similar baseline HAI 270 titers from participants” perhaps say similar TO?

**Author response:** We have revised the sentence. Line 304-307

**Minor comment:**

Line 263. The heat map is a good idea. Shouldn’t this explanation be, at least in part, in the legend? What does the blue column on th right (H3N2) mean? It appears to mean participants are ordered by decreasing titer, but they don’t seem to be.

**Author response:** Thank you for this suggestion. We have revised the Figure 1 legend to clarify the heat map encoding and layout. Specifically, we now state that the “blue column” at the far right is the color scale bar (key) indicating log₂ HAI titers and is not an additional data column or a participant-ordering key. We also clarified the ordering of participants on the y-axis: participants are grouped by age category (birth year; youngest to oldest) and are not ranked by decreasing HAI titer within age strata. These additions have been incorporated into the revised Figure 1 legend to prevent misinterpretation. Please find figure 1 legend in between Line 284-301.

**Minor comment:**

LINE 285. “ Fewer participants between 18–34 years old had HAI titers >1:40 at D0 during the 2022-2023 season compared the 287 next two seasons, but had HAI titers >1:40 following vaccination on D28, with modest back288 boosting against historical H3N2 isolates. The point of the second part of the sentence is not clear—is a word missing??

**Author response:** We revised the sentence to clarify the intended contrast (baseline D0 versus post-vaccination D28) and to correct the grammar. Line 322-325.

**Minor comment:**

Line 480 and following and Table 4. Is this referring only to the final season of the study? How do 2017-2019 come into it (footnote to Table 4)? Line 485…which participants are meant by “these participants”? Line 490 and following is very hard to follow. Should it say …participants WHO were not……? Is the B Victoria component of the vaccine influencing the B Yamagata response? Please comment in Discussion section.

**Author response: Line 480 and Table 4:** Table 4 and the text refer to participants vaccinated in the final season (2024–2025; UGA9), stratified by whether they received influenza vaccination in the two preceding study seasons (2022–2023 [UGA7] and 2023–2024 [UGA8]). We revised the text and table caption/footnote to address this concern. The reference to 2017–2019 in the Table 4 footnote has been corrected. The footnote now defines “prior two seasons” strictly as the two preceding study seasons (UGA7 and UGA8). We also clarified the referent by explicitly stating whether we are describing participants seroprotected at baseline (D0 ≥1:40) versus seronegative at baseline (D0 <1:40), eliminating ambiguous phrasing. Line 537-545.

**Line 490:** We rewrote the sentences from Line 548 onward, including correcting the phrasing to “participants who were not vaccinated…” and restructuring the comparisons by baseline serostatus (D0).

During preparation of the revised manuscript, we identified discrepancies in Table 4 that occurred during manual transfer of values from the Excel analysis file into the Word table. As a result, some values in the original table were reported incorrectly and the corresponding interpretation required revision. We have now carefully rechecked the underlying dataset, regenerated Table 4 from the verified data, and revised the related sections of the Results and Discussion to ensure full consistency with the corrected values. We apologize for this oversight and for any confusion it may have caused (Lines 508–570). All data used in this manuscript now have been supplemented.

**B/Victoria vs B/Yamagata:** We briefly discussed clarifying that any changes in B/Yamagata HAI titers in 2024–2025 are interpreted as pre-existing immunity/back-boosting and/or variability, and cannot be attributed directly to the B/Victoria vaccine component. Line 853-855.

**Minor comment:**

Line 530… those who switched from SD to…word(s) missing ???

**Author response:** Thank you for catching this omission. We have corrected line 530 to read: “…… those who switched from SD to HD across the study period”. Line 606-607.

**Minor comment:**

It’s hard at first to see what’s different between two paragraphs in results describing Figs 6 and 7, and the two figures. Titles emphasizing IAV and IBV would help.

**Author response:** We have added subheadings to clearly distinguish between the influenza A and B analyses and their corresponding figures. Line 583 and Line 608.

**Minor comment:**

Line 668-671. ….’a smaller proportion had detectable titers and achieved…. “ Possibly this could be more clearly stated as ‘ a smaller proportion fell into the group that initially had detectable titers and then achieved..”

**Author response:** Thank you for this suggestion to improve clarity. We have revised the sentence. Line 771-772.

**Minor comment:**

Line 714. A D amino acid sounds like chirality is being discussed. ‘..had the amino acid D’ might be clearer.

**Author response:** Thank you for identifying this ambiguity. We have revised the text to be clearer. Line 822.

**Minor comment:**

Is it correct that titers to B Yamagata rose during the 2024-25 season even though it was not in the vaccine? Could this be because of a few outliers who got infected with B Yamagata and raised the average or extreme cross-boosting?

**Author response:** In the discussion, we interpreted that these antibodies had persisted into the 2024-25 season despite the vaccine no longer containing the B/Yamagata component instead of rise in HAI. We have now revised the text by stating that there was measurable seroconversion in some participants, and we revised the results accordingly to address this confusion. This observation couldn’t be due to outliers because 14.69% (72/490) of participants showed seroconversion against Yamagata in the 2024-25 season, compared with 38.62% and 18.04% in the previous seasons 2022-23 and 2023-24, respectively. Line 835-855.

**Minor comment:**

Line 737 ‘and thereby avoidS…

**Author response:** We have corrected subject–verb agreement (“avoids”). Line 861.

**Minor comment:**

Line 738… while this statement (may avoid mismatches with circulating strains due to egg adaptation) may be correct, does it explain your results? Methods states you grew the indicator viruses for the HAI assays in eggs? (Was that a disadvantage for FluBlok and FluCelVax measured titers? Please comment.

**Author response:** We agree that performing HAI assays with egg‑propagated indicator viruses can theoretically favor egg‑based vaccines, since antibodies elicited by egg‑adapted HA may recognize egg‑grown assay viruses more efficiently than antibodies induced by cell‑based or recombinant formulations. In response, we have added text to the Discussion acknowledging this as a limitation of our study and clarifying the potential bias. Importantly, however, Flublok elicited significantly higher HAI titers than the egg‑based comparators in younger adults, and Flucelvax generally performed comparably to egg‑derived vaccines, even under these assay conditions. Thus, any bias introduced by the use of egg‑grown indicator viruses would tend to underestimate, rather than exaggerate, the relative immunogenicity of the cell‑based and recombinant platforms. We now explicitly state that our findings for Flublok and Flucelvax should therefore be interpreted as conservative estimates of their immunogenic advantage. Please see lines 876-884.

**Minor comment:**

735 and following—please clarify whether your data sheds any light on the relative value of Flublok vs FluAd and/or Fluzone HD in older adults.

**Author response:** Our study was not designed to directly compare Flublok to Fluad or Fluzone HD in adults ≥65 years because Flublok was indicated for and administered primarily to younger adults (18–64 years), whereas Fluad and Fluzone HD were indicated exclusively for the elderly (≥65 years). While a few Flublok recipients (n=8) were aged 65–79 (Table 1, Fig 9), the sample size in this stratum was insufficient for robust statistical comparison, and the substantial age difference between cohorts (mean age ~44 years for Flublok vs. ~73–74 years for Fluad/Fluzone HD) confounds any indirect comparison. Consequently, our data cannot determine whether Flublok offers superior, equivalent, or inferior immunogenicity compared to the enhanced formulations in older adults, and the text has been revised to clarify this limitation explicitly. This can be found in lines between 871 and 874.

**Minor comment:**

748 and following—it might be worth looking at titers in pediatric participants alone. Also, perhaps mention that half the age span of pediatric patients in general was omitted from this study. Younger children’s responses probably differ.

**Author response:** Thank you for highlighting the pediatric age limitation. We have added the following text to the Discussion acknowledging this study design choice:

"Notably, the pediatric age span (birth to 8 years) was not included in our study, as younger children often exhibit distinct immune responses to influenza vaccination due to differences in immune maturation, lower baseline immunity, and potentially higher reactogenicity." Lines 893-896.

**Minor comment:**

Line 762. “In contrast, participants in all age groups had (descriptive word missing?) HAI titers against the two influenza B vaccine components compared to HAI titers against influenza A vaccine components.”

**Author response:** Based on the data presented in Figures 6 and 7, participants maintained higher HAI titers against influenza B compared to influenza A. We have corrected missing descriptive word “higher” now. **Line 902.**

**Reviewer #1: Reviewer Comments:**

Manuscript Number: PONE-D-25-48769

Title: Assessment of Hemagglutinin-Inhibition Activity Following Influenza Vaccination During the 2022-2023, 2023-2024, and 2024-2025 Seasons.

This is a 3-season report on the immunogenicity of six influenza vaccine types in 1328 participants aged 9-89 years old enrolled in an observational cohort study, 203 of whom received an influenza vaccine in all 3 seasons allowing longitudinal assessment. Overall, the manuscript is well written, however there are a few major and multiple minor revisions needed.

**Major Comment 1:**

1. Page 21, Lines 165-167: HAI titers of ≥40 are considered non-detectable or seronegative in this manuscript, which is incorrect as the lower limit of detection of HAI test is a dilution of 1:10, and <10 is considered not detectable or seronegative. Also, a titer of ≥40 or more is considered seropositive or seroprotection (if it is a post-vaccination titer), but seroprotection and seropositivity are used interchangeably on D0 in the figures. Consider using alternative terminology such as no/low HAI titer <40 vs. HAI titer ≥40. Other alternatives are using no-seroprotection vs. seroprotection. The definition of seroconversion looks good.

**Author response:** We thank the reviewer for their careful examination of our manuscript. We have corrected the seroprotection/seronegative terminology to clearly distinguish between "non-seroprotective" (<1:40), and "seroprotective" (≥1:40) throughout the text.

**Major Comment 2:**

Page 22, Line 195: Regarding comparisons between vaccine types, Dunnett’s multiple comparison test is appropriate if there is one control group and the one-way ANOVA is significant. If there are multiple comparison groups, then Tukey or Scheffe test may be more appropriate.

**Author response:** We thank the reviewer for this important comment. In response, we re-analyzed the between-vaccine comparisons using Games-Howell’s multiple comparisons test rather than Dunnett’s T3. Although Tukey or Scheffé tests can be appropriate in some ANOVA settings, Games-Howell was more suitable for our dataset because it accommodates unequal variances and unequal sample sizes, both of which were present in our analyses (Brown-Forsythe test, p < 0.0001 for all components; for example, Fluzone SD n = 453 vs. Flublok n = 102). Reanalysis with Games-Howell did not change the primary conclusions: Flublok remained significantly superior to the other vaccines across all four influenza components, the overall ranking of vaccine immunogenicity was preserved, and the H3N2 results showed the same compact letter groupings. We have updated the Methods accordingly to state: “Between-vaccine comparisons were evaluated using one-way ANOVA with Games-Howell’s multiple comparisons test.”. (Line 224, Methods; Figure 8 legend, Line 651-664)

**Major Comment 3:**

3. Page 24, Line 245: Supplemental Table 1 and text have discrepant vaccine types – e.g. line 245 (FB>FB>FB; n=16) whereas Supplemental Table 1 shows FM>FM>FM, n=16 and does not show anyone with FB>FB>FB. Also, this Table shows FB > FC > FB = 8 in row 6 from top and again FB > FC > FB = 1 four rows up from the bottom Total row. Similarly, FZ SD >FZ HD > FZ HD are listed twice with N = 5 and 1 each.

**Author response:** Thank you for identifying these discrepancies. We have corrected Supplemental Table 1 as follows: Removed duplicate entries for FB > FC > FB: Consolidated the duplicate rows (previously showing n=8 and n=1) into a single entry with the correct total of n=9.

Removed duplicate entries for FZ SD > FZ HD > FZ HD: Consolidated the duplicate rows (previously showing n=5 and n=1) into a single entry with the correct total of n=6. Please see S1 table I supplement data and line 272-276 I in the revised text.

**Minor Comments**

**Minor Comment 1:**

1. Page 11, Line 59: Please clarify if the lower vaccine-induced response is meant for standard-dose inactivated vaccine.

**Author response:** We have clarified the text to specify that lower vaccine-induced immune responses in older adults refer specifically to standard-dose inactivated vaccines. This distinction is supported by both cited references: Lang et al. (2012) and Domínguez et al. (2016). Please see Line 62.

**Minor Comment 2:**

2. Page 12, Line 89: Please clarify if individual vaccinees with more than one dose in a study season were excluded or not.

**Author response:** We have clarified concurrent participation to another influenza vaccine studies are excluded. Line 97-98.

**Minor Comment 3:**

3. Page 19, Line 121, Table 3: Please clarify with a footnote when the age group and BMI are for individuals in Table 3. ?First vaccination, last vaccination or some other time.

**Author response:** We have clarified age and BMI variables represent first enrollment during 2022-2023 season. Please find Line 143, Footnote of Table 3.

**Minor Comment 4:**

4. Page 21, Line 173. Please spell out what BEI stands for.

**Author response:** We have spelled out BEI now in the text and stand for Biodefense and Emerging Infections. Line 201.

**Minor Comment 5:**

5. Page 23, Lines 212-213: The proportion of adolescents (≤17 years) was relatively small in the 2022-2023 season (9.5%) and 2024-2025 season (5.3%) but increased to 6.5% in 2023-2024 season. Please correct this sentence because the proportion of children <18 years decreased from 9.5% to 6.5% to 5.3% in the 3 consecutive study seasons.

**Author response:** We agree and we have revised the sentence as follow: The proportion of adolescents (≤17 years) was relatively small across all three seasons compared to other age groups, decreasing from 9.5% in 2022-2023 to 6.5% in 2023-2024 and further to 5.3% in 2024-2025. Line 240-242.

**Minor Comment 6:**

6. Page 23, Lines 216-218: Consider deleting this sentence here because supporting data is shown later under vaccination patterns in repeaters.

**Author response:** We have removed this sentence as requested. **Line 246, removed.**

**Minor Comment 7:**

7. Page 25, line 267: Figure 1A Y-axis label shows ages 10-17 years instead of 9-17 years.

**Author response:** We have corrected Fig 1A Y axis label now. Please see revised fig 1.

**Minor Comment 8:**

8. Page 25, line 276: There is no titer done at 6 months from the pre-vaccination baseline so it might be better to say that the titer had waned prior to vaccination next season rather than wanes during the season.

**Author response:** We agree on this comment, we have revised text addressing the comment: “Following vaccination, there were strain-matched rises in HAI titers, but a more limited increase in cross-reactive HAI titers that had waned by the subsequent season's baseline.” Lines 312-315.

**Minor Comment 9:**

9. Page 25, lines 283- 285: There were no titers done 365 days after 2024-25 season and DR21 titers seem to have waned by 2024-25 baseline, please clarify this sentence.

**Author response:** We agree with this comment, we have revised the paragraph avoiding 365 days after 2024-25 season and clarified titer wane by 2024-2025 for DR/21. **Line 322.**

**Minor Comment 10:**

10. Page 28, line 361: Statistically “similar” or “significant”?

**Author response:** Thank you for your attention. The values are statistically similar between D0 and D28; however, we have corrected the season to 2024–2025. **Please see 406-407.**

**Minor Comment 11:**

Page 28, line 378: Clarify seroconverted against A (H1N1).

**Author response:** Thank you we have clarified this now in the text. Line 428.

**Minor Comment 12:**

Page 28, line 384: Is the 28% B/Yamagata seroconversion correct for average over 2 seasons ...

**Author response:** Yes, 28% B/Yamagata seroconversion rate represents the average of the two seasons when this lineage was included in the vaccine (2022-2023: 38.62%; 2023-2024: 18.04%). The 2024-2025 season is excluded as B/Yamagata was not a vaccine component that season. Line 435-36.

**Minor Comment 13:**

Page 29, Figure 4, line 401. There are no numbers seen on the X-axis in this figure, it shows the seasons.

**Author response:** We have revised figure legend, and added the sample sizes (n=378, n=460, n=490) to the figure legend instead showing on the figure. Line 392-93.

**Minor Comment 14:**

Page 30, line 418: Re ages 18-34 years, should this be 35-49 years?

**Author response:** We agree, you are correct. We have revised the text to accurately reflect the data. The corrected sentence now reads: “During the 2024-2025 season, participants 50-64 and ≥65 years old had the highest seroconversion rates (**~** 25%) and participants 35–49 years had the lowest (~19%) seroconversion rates (Fig 5A-5C)”. Please see lines 468 to 471.

**Minor Comment 15:**

Page 30, line 420: Re 33-53% against A (H3N2)? Please clarify.

**Author response:** We have checked and clarified these data against H3N2. Now it has been corrected. Line 471.

**Minor Comment 16:**

Page 30, lines 428-429, re B/Vic: Oldest participants show higher seroconversion than youngest in 2 of 3 seasons.

**Author response:** We checked and revised this. It reads now: “In general, younger participants had higher non-seroprotective to seroprotective rates than older participants for H3N2 across all seasons and against B/Yamagata in 2022-2023 and 2023-2024 seasons, whereas H1N1 rates varied by season without consistent age-related trends, and B/Victoria conversion was often higher in older participants (≥50 years) compared to younger participants (≤34) (**Fig 5**).”. Please see lines between 480 and 485.

**Minor Comment 17:**

Page 30, lines 434-435: Number of participants not seen along the X-axis in Fig 5.

**Author response:** We have revised the Figure legend reflecting the Figure now and number of participants are provided in the legend. Line 488-505

**Minor Comment 18:**

Page 30, lines 434-435: Consider replacing UGA9 with 2024-2025 season.

**Author response:** Thank you for suggestion, we have now replaced UGA9 with 2024-2025 season. Line 503.

**Minor Comment 19:**

Page 32, Table 4, line 478: Seasons listed need corrections.

**Author response:** Thank you for this. We have corrected Table 4 and the corresponding figure legends to reflect 2024–2025 data only. In addition, we corrected Table 4 in its entirety. We identified discrepancies in Table 4 that occurred during transferring data from Excel into the Word table, which resulted in some incorrect values and, consequently, incorrect interpretations. We have now revised the Results and Discussion sections related to Table 4. Overall conclusions still holds, but values and some rates are changed. We apologize for this error and for any inconvenience it may have caused (Please see corrected and revised results in Lines 508–570 and Table 4, we have provided raw data in supplementary files S6 Data in Table 4, UGA9_2024_2025 tab).

**Minor Comment 20:**

Page 32, Table 4, line 479: It is not clear if the Fisher’s exact test was used for this table ...

**Author response:** We have updated Tale 4 with revised data. Fisher test no longer required and we have revised the legend. Line 538-546.

**Minor Comment 21:**

Page 33, line 493: Correction: 30% did seroconvert. Delete not at the end of the sentence.

**Author response:** We have revised the text now. The corresponding paragraph has been revised entirely with corrected data. Line 563-564.

**Minor Comment 22:**

Page 35, line 556, consider naming the six vaccines here.

**Author response:** We have now revised and included all vaccine names: “Participants vaccinated with any of the six vaccines (Fluzone Standard Dose, Fluzone High-Dose, Fluad, Flublok, Flucelvax, and Flumist) had, on average….” Lines 636-637.

**Minor Comment 23:**

Page 36, line 588: Correction - 2023-2024 season (Fig 9B) compared to 202-23 season (Fig 9A).

**Author response:** Thank you, we have made corrections. Lines 669-670

**Minor Comment 24:**

Page 37, Fig 9, line 619: Red Asterix is not seen.

**Author response:** We have revised the text, no red asterisk is shown in figures. Line 701-703.

**Minor Comment 25:**

Page 38, line 645 – correction, to historical strains rather than to drifted viral variants.

**Author response:** We made revision to historical strains. Line 744.

**Minor Comment 26:**

Page 39, line 662, consider saying to previous two years rather than 1-2 years.

**Author response:** Thank you, we have revised the text: “…influenza vaccination in the previous two years had higher overall seroconversion rates…” Line 764.

**Minor Comment 27:**

Page 39, line 669, change detectable to protective or whatever alternative terminology is used, as appropriate.

**Author response:** We have revised text as requested: “…a smaller proportion achieved such rises compared to those without prior vaccination….” Line 771-72.

**Minor Comment 28:**

Page 41, line 735, specify participants age range for Flublock

**Author response:** We have revised the text by indicating age range in the text: “Participants aged 18-79 years vaccinated with Flublok had the strongest serum HAI activity following vaccination”. Line 860

**Minor Comment 29:**

Page 42, line 770: to maintain ‘seroprotection or HAI titers ≥40’.

**Author response:** Thank you for this suggestion, we have added this to the end of our sentence: “….formulations and/or booster strategies to maintain seroprotection or HAI titers ≥40.” Line 909-910.

Sincerely,

Ted M. Ross, Ph.D.

Director – Global Center for Vaccine Development, - Department of Infection Biology

Cleveland Clinic Florida

Research and Innovation Center
